# Supplementary material for: Causal Relationship between Aging and Anorexia Nervosa: A White-Matter-Microstructure-Mediated Mendelian Randomization Analysis
Source: Biomedicines. 2024 Aug 16;12(8):1874. doi: 10.3390/biomedicines12081874 (PMC11351342; doi:10.3390/biomedicines12081874)
Supplement: Supplementary file 1 [file biomedicines-12-01874-s001.zip › Supplementary Figure/Figure S7/Single SNP plot of MR analysis for ubm-b-1624 to AN.pdf]

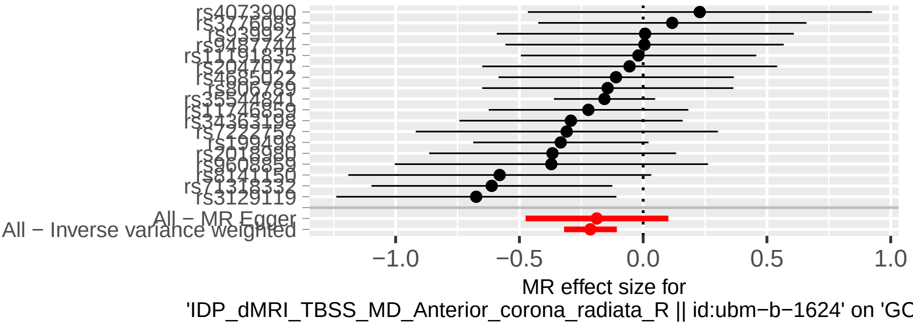
rs4073900
rs3776089
rs939924
rs9487744
rs11191835
rs2047071
rs4685022
rs806789
rs35544841
rs11746859
rs34363198
rs7222757
rs199498
rs2018980
rs9608859
rs8141150
rs71318332
rs3129119
All − MR Egger
All − Inverse variance weighted
−1.0
−0.5
0.0
0.5
1.0
MR effect size for
'IDP_dMRI_TBSS_MD_Anterior_corona_radiata_R || id:ubm−b−1624' on 'GC
